# Endorectal Ultrasound (ERUS): an Accurate and Invaluable Tool for Identifying Suitable Candidates for Transanal Excision/Transanal Endoscopic Microsurgery (TAE/TEM) in Early-Staged Rectal Tumors

**DOI:** 10.1007/s11605-023-05868-6

**Published:** 2023-11-06

**Authors:** Charlotte Campbell, Jason Conway, Laura Elizabeth Lavette, Darius Jahann, Gregory Waters, Jean Ashburn, Girish Mishra

**Affiliations:** Atrium Health Wake Forest Baptist Medical Center, Medical Center Blvd, Winston-Salem, NC 27157 USA

**Keywords:** Endorectal ultrasound, Staging, Transanal excision/transanal endoscopic microsurgery

Colorectal cancer is the third-most common cancer diagnosed in the USA.^1^ Surgical resection of disease remains the mainstay of curative therapy. Efforts to improve morbidity have focused on minimally invasive procedures such as transanal excision (TAE) and transanal endoscopic microsurgery (TEM) for early-stage lesions. Differentiating early-stage lesions that can be resected with minimally invasive techniques from locally advanced cancers that require radical resection (with its inherent morbidity and mortality) is critical.^2, 3^ We hypothesized that endorectal ultrasound (ERUS) can accurately discriminate early versus advanced neoplastic lesions, especially in low-lying rectal tumors that can achieve R0 resection via TAE/TEM.

**Fig. 1:**
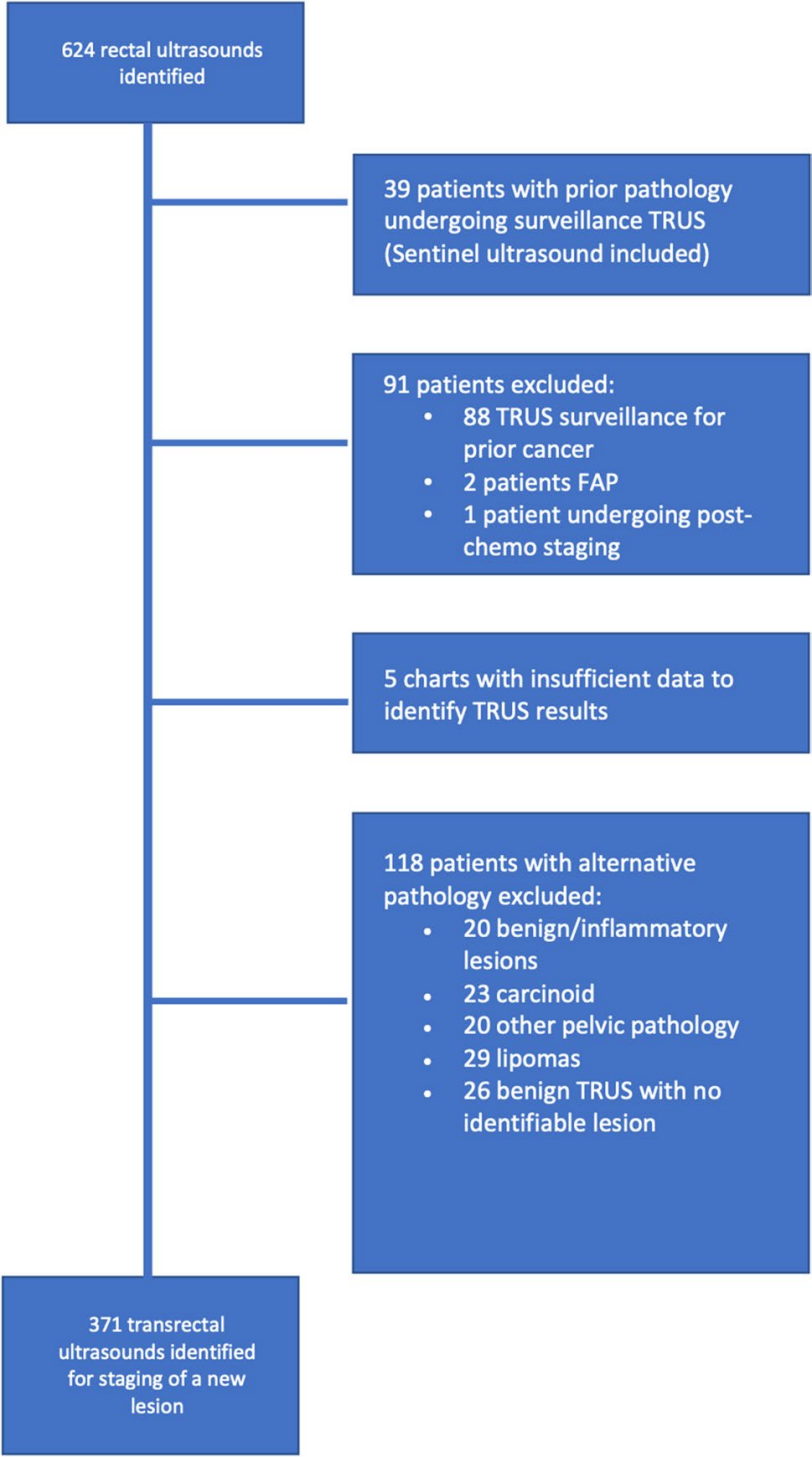
Schema for patient inclusion and exclusion criteria

We extracted data on all patients undergoing ERUS between January 2011 and December 2020. All staging ERUS were performed using the Olympus GF-UE 160-AL5 radial echoendoscope. Patients with adenomatous polyps, carcinoma in situ, or adenocarcinoma on pathology were included; other patients with benign pathology, surveillance ERUS, or non-carcinomatous malignancy were excluded (see Fig. 1).

In total, 371 patients with qualifying criteria were identified (median age = 64; range = 19-96) with the average lesion size 3.76 cm [0.3–15.0 cm]. Postoperative pathology was available on 175 patients of the 264 patients who were found to undergo surgical procedures. Test characteristics for ERUS are presented in Table 1 and include the following: Agreement = 0.90; Sensitivity = 0.96; Specificity = 0.84; PPV = 0.88; NPV = 0.94, with no differences based on gender, age (<65 vs 65+) or lesion size (<3.0 cm vs 3+ cm).

**Table 1:** Test characteristics of ERUS

	*N*	Sensitivity	Specificity	PPV	NPV
Overall	175	0.96	0.84	0.88	0.94
Gender					
Female	73	0.97	0.86	0.88	0.97
Male	102	0.95	0.82	0.87	0.92
Age					
<65	87	0.98	0.76	0.87	0.96
65+	88	0.93	0.89	0.89	0.93
Lesion size					
<3 cm	62	0.94	0.92	0.94	0.92
3+ cm	87	0.95	0.82	0.83	0.95
Procedure					
TAE/TEM	92	0.90	0.98	0.97	0.95
LAR	60	0.98	0.50	0.89	0.86
APR	21	1.0	0.0	0.71	--

In our study, over 98% of patients staged T0/Tis were <T2. Furthermore, no patients undergoing an APR had T0/Tis disease. Very early studies performed by Hildebrandt and colleagues similarly showed that the most significant impact of endosonography was that the proportion of abdominoperineal excisions dropped from 46 to 15% over a 5-year period.^4^ We report phenomenal success using ERUS in determining which patients should undergo a TAE/TEM procedure (PPV and NPV = 0.97 and 0.95 respectively). Our study has several limitations including the following: (1) it was a single-center trial performed at a tertiary referral center with endoscopists highly specialized in performing ERUS and (2) most patients in this study did not have MR data to create additional conclusions about direct comparisons of the tools’ efficacies.

Our study primarily looked at the overstaging and understaging of mucosal- vs submucosal-based lesions and does not address the complexities of distinguishing T1 and T2 lesions. We were highly proficient in not overstaging, as no patients underwent an APR based on our ERUS findings — this point alone is of great solace to our patients and management team so as to avoid the untoward challenges of a more morbid and invasive APR. Given its ability to identify early rectal cancer, we exhort our surgical colleagues to not abandon ERUS as it remains a wonderful, additive tool to MRI for properly identifying these lesions most amenable to minimally invasive surgery.
